# Symmetric three-port laparoscopic Roux-en-Y gastric bypass: a novel technique that is safe, effective, and feasible

**DOI:** 10.1007/s00595-022-02629-x

**Published:** 2023-02-04

**Authors:** Biao Zhou, Xinyu Cao, Zhe Wang, Nianrong Zhang, Baoyin Liu, Hua Meng

**Affiliations:** 1 Department of General Surgery & Obesity and Metabolic Disease Center, China-Japan Friendship Hospital, Beijing, 100029 China; 2 Institute of Clinical Medical Sciences, China-Japan Friendship Hospital, Beijing, 100029 China

**Keywords:** Symmetric three-port laparoscopic Roux-en-Y gastric bypass (STLGB), Single-incision laparoscopic surgery (SILS), Weight loss, Obesity

## Abstract

**Purpose:**

Single-incision laparoscopic surgery (SILS) has been validated as a safe approach for bariatric surgery. However, as the utilization of SILS in bariatric surgery is still limited by its disadvantages, this study analyzes the outcomes of symmetric three-port laparoscopic Roux-en-Y gastric bypass (STLGB).

**Methods:**

The medical records of patients who underwent STLGB between January 2018 and February 2021 were analyzed retrospectively using an institutional database. The patients were divided into four groups according to their baseline body mass index (BMI). The primary endpoints were operative time, length of stay, complication rate, and weight loss 12 months after surgery.

**Results:**

We analyzed the records of 101 patients who underwent STLGB. There was a slight predominance of women (*n* = 61; 60.4%). The mean operative time was 97.16 ± 38.79 min and the length of stay in the hospital after surgery was 2.79 ± 1.4 days. One patient (0.99%) suffered a gastrojejunal anastomosis leak within 30 days of surgery. There were no significant differences in LOS, complication rate, or cosmetic score among the four groups. The mean BMI reduction was 8.67 kg/m^2^ and the % total weight loss (%TWL) was 24.37%. Weight loss measured 12 months after surgery was significantly different among the four groups.

**Conclusions:**

STLGB is safe, effective, and feasible for all kinds of patients. It is reproducible with standardization of the procedure.

## Introduction

Bariatric surgery has proven to be the most effective treatment for morbid obesity and its associated comorbidities, including type 2 diabetes mellitus (T2DM), hypertension, dyslipidemia, nonalcoholic fatty liver disease (NAFLD), obstructive sleep apnea (OSA), and other diseases [1, 2]. The laparoscopic approach benefits both the patient and the surgeon in several aspects, including minimized blood loss, low complication rates, less postoperative pain, and a shorter hospital stay.

Single-site or single-incision laparoscopic surgery (SILS) has been used in several types of surgery, including gynecologic, urological, and gastrointestinal operations [3–5]. Through a single umbilical incision, the same operation is performed with better cosmetic results since no additional incisions are made in the abdominal wall, leaving no visible abdominal scars [6]. Reports have been published in the last decade of SILS being applied for different bariatric surgeries, such as laparoscopic adjustable gastric banding (LAGB), laparoscopic sleeve gastrectomy (LSG), and laparoscopic Roux-en-Y gastric bypass (LRYGB) [7–9]. Single-incision laparoscopic bariatric surgery has been shown to be associated with less postoperative pain, a shorter hospital stay, and a lower analgesic dosage than conventional laparoscopic surgery [10].

LRYGB is a complex type of bariatric surgery with a steep learning curve. Conventional laparoscopy requires five to seven abdominal incisions; however, it is difficult for most bariatric surgeons to perform single-incision LRYGB, and its application is highly limited by patient conditions. Most of these reports described elective surgeries with exclusion criteria such as a BMI > 50 kg/m^2^, xiphoid-umbilical distance (XUD) > 28 cm, revision surgery, or previous abdominal surgeries [11]. Single-incision LRYGB has not been recommended for routine use because of these disadvantages. In the present study, we describe a symmetric three-port laparoscopic RYGB (STLGB) technique that could become a routine procedure. To evaluate this technique, we collected data from 101 consecutive patients who underwent STLGB.

## Methods

Between January 2018 and February 2021, 101 consecutive patients underwent STLGB at our center. The institutional ethics committee of our hospital approved the study, and written informed consent was obtained from each patient. All patients were advised by a multidisciplinary team about the benefits, risks, and long-term outcomes of the procedure. All operations on all 101 consecutive patients were performed by one surgeon. This was a retrospective analysis of electronic data, collected prospectively, on bariatric surgery.

Following the latest guidelines for metabolic surgery from the Chinese Society for Metabolic and Bariatric Surgery (2019), the inclusion criteria were as follows: patients who were overweight (25 ≤ BMI < 27.5 kg/m^2^) and had poorly controlled type 2 diabetes mellitus (T2DM), despite fully optimized conventional therapy with either oral or injectable medications; patients with T2DM and obesity (BMI ≥ 27.5 kg/m^2^) with inadequately controlled hyperglycemia despite lifestyle and optimal medical therapy; and patients with super obesity and severe gastroesophageal reflux disease (GERD) without T2DM. Patients who underwent revision surgery were excluded.

The patients were divided into four groups according to their body mass index: Group I (G I): 25 ≤ BMI < 27.5 kg/m^2^; Group II (G II): 27.5 ≤ BMI < 32.5 kg/m^2^; Group III (G III): 32.5 ≤ BMI < 37.5 kg/m^2^; and Group IV (G IV): BMI ≥ 37.5 kg/m^2^. BMI was categorized according to the American Diabetes Association (ADA) guidelines and the Chinese Society for Metabolic and Bariatric Surgery guidelines [12].

## Operative technique

### Skin incision and trocar placement

**Fig. 1:**
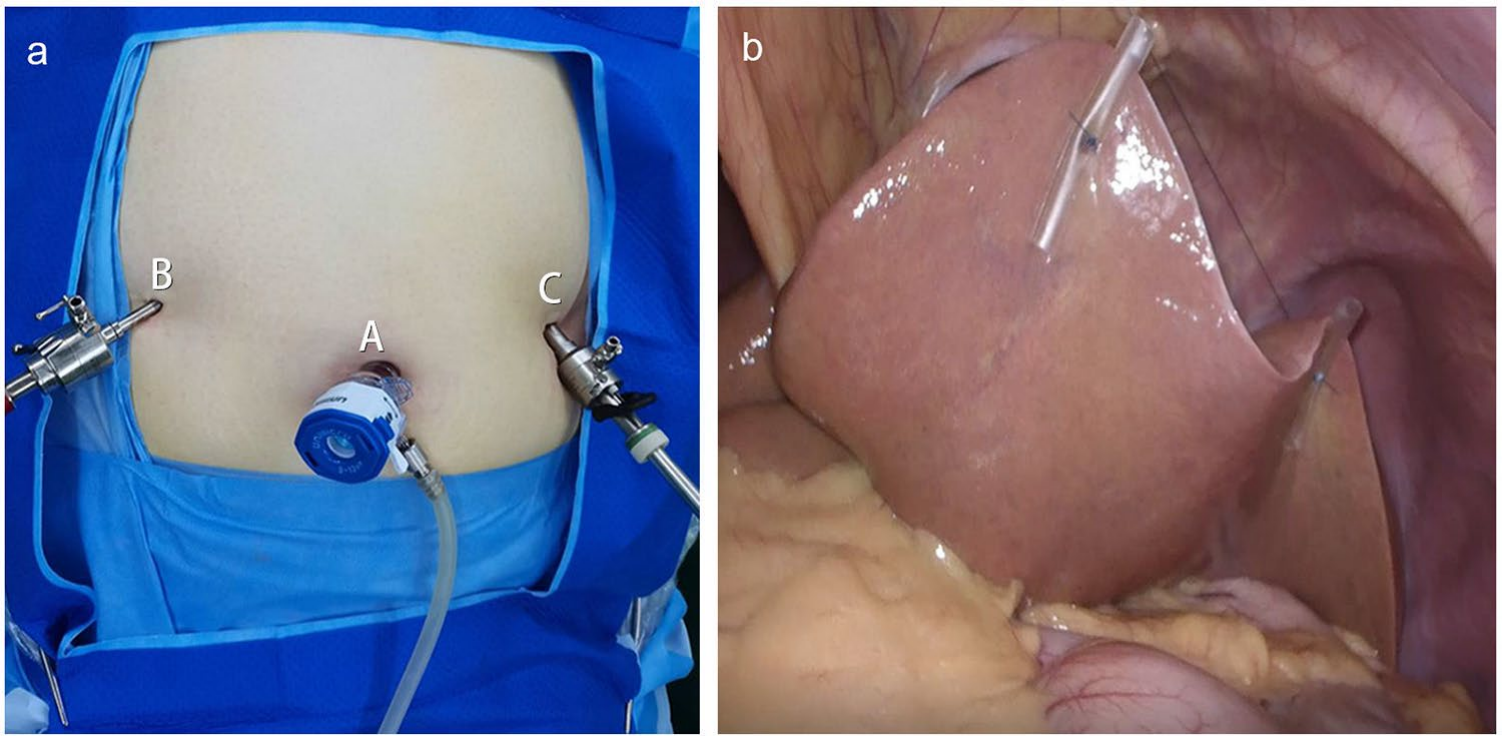
**a** Three symmetrical ports form an inverted isosceles triangle (12 mm in the umbilicus, 10 mm in the left lateral abdominal wall and 5 mm in the right lateral abdominal wall). **b** The liver is penetrated by two straight needles and retracted upward with sutures, fixed using Kelly clamps

The patient was placed supine with their arms extended laterally and their legs together. The surgeon stood on the right side of the patient and the assistant stood on the left. Three skin incisions were made in the abdomen: a 12 mm vertical incision at the umbilicus for the working channel, a 10 mm incision in the upper left aspect of the abdominal wall on the left mid-clavicular line for the video scope, and a 5 mm incision in the upper right aspect of the abdominal wall symmetrical with a 10 mm hole on the right mid-clavicular line as the secondary operation site (Fig. 1a). In patients with a shorter XUD, the two symmetrical incisions were lower, possibly even at the umbilical level. A 30°, 10 mm laparoscope was inserted through the observation incision to obtain an adequate surgical field of view. CO_2_ was then insufflated until the pneumoperitoneum pressure reached 14 mmHg. Subsequently, 2–0 polyamide sutures were placed around the middle of the drainage tube, and the two straight needles attached to the sutures were used to retract the left lobe of the liver to obtain a clear operative field (Fig. 1b).

### Symmetric three-port laparoscopic gastric bypass surgery (STLGB)

The operation was started by dissecting a window in the lesser curvature of the gastric body with an electric hook. The site of dissection along the lesser curvature for pouch creation was just proximal to the second branch of the left gastric vessel. We created a retro gastric tunnel by blunt dissection using bowel forceps. After creating the retro gastric tunnel, the first stapler was fired horizontally using 60-mm linear cutting staplers (ECS-60-L(R), WASTON, China) with a blue cartridge (ECZ-60-B, WASTON, China), followed by two vertical firings to create a gastric pouch of 15–20 mL. A bougie was not used when a gastric pouch was created. After measuring 100 cm below Treitz’s ligament, a small incision was made in the small intestine using the monopolar hook. An articulator 60 mm linear cutting stapler with a blue cartridge was applied to the alimentary incision to create the gastrointestinal anastomosis. The afferent loop was transected using the white stapler and a stapled side-to-side jejunoileal anastomosis was created. The biliopancreatic limb was 50 cm long for patients with a BMI of 25–27.5 kg/m^2^ and 100 cm long for patients with a BMI of > 27.5 kg/m^2^. The gastrojejunal anastomosis was closed by a hand-sewn technique and mesenteric defects were closed using unabsorbable sutures. The three incisions were closed with 3–0 absorbable bidirectional sutures (Ethicon, USA).

### Postoperative care

All patients received standard postoperative care under an enhanced recovery after surgery (ERAS) pathway. Follow-up 1, 3, and 6 months, and then 1 year after discharge was advised for all patients.

### Cosmetic evaluation

**Fig. 2:**
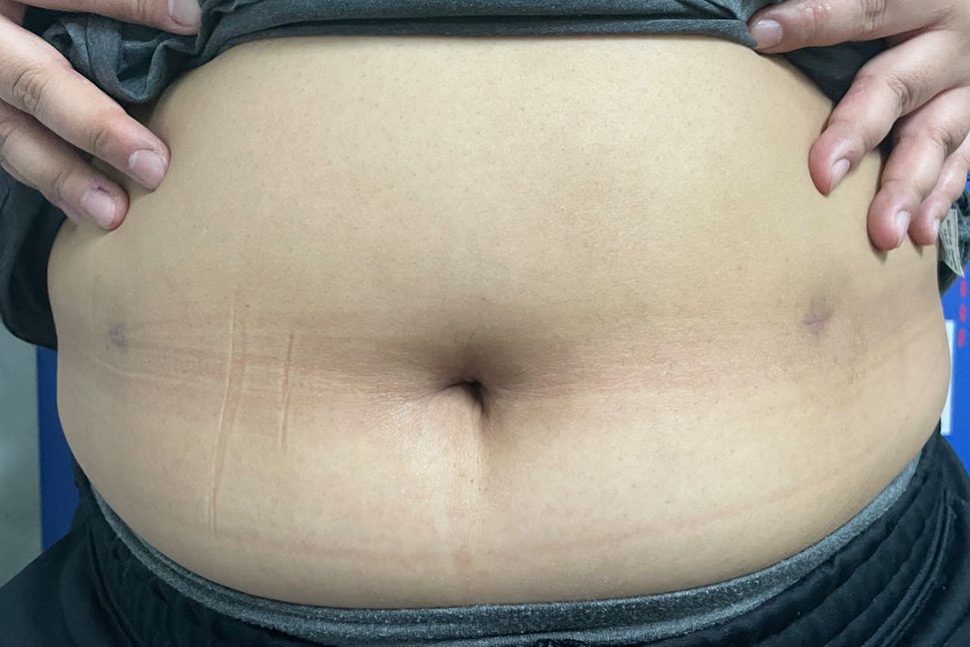
Cosmetic appearance of the wounds 1 month after surgery

We evaluated, retrospectively, the cosmetic results of the postoperative wounds 1 month after surgery (Fig. 2). The patients’ satisfaction with the wound was measured using a scoring system ranging from 1 (very unsatisfied) to 5 (very satisfied) [13].

### Statistical analysis

Continuous variables are expressed as means ± standard deviation (SD), whereas categorical variables are expressed as numbers and percentages. All statistical analyses were performed using SPSS statistical software (version 22.0; USA). One-way analysis of variance was used to compare the continuous variables among the four groups. Student’s t-test was used to compare the weight and BMI between baseline and follow-up. Significance was defined as *P* < 0.05.

## Results

A total of 101 consecutive STLGB procedures were performed. The mean age of the patients was 42.18 ± 9.44 years and the majority were women (*n* = 61; 60.4%). The preoperative mean weight was 94.20 ± 21.29 kg and the mean BMI was 33.59 ± 6.68 kg/m^2^ (Table 1). The most common comorbidity among the patients was T2DM (91.1%), followed by hypertension (29.7%), hyperlipidemia (29.7%), and obstructive sleep apnea (5%). An extra port was required to complete the procedure in three patients (2.7%) because of adhesions.

**Table 1:** Demographic characteristics of the study patients

Patient characteristic (*n* = 101)	
Gender (F), *n* (%)	61 (60.4%)
Age (y), mean ± SD	42.18 ± 9.44
Weight (kg), mean ± SD	94.20 ± 21.29
BMI (kg/m^2^), mean ± SD	33.59 ± 6.68
Operative time (min), mean ± SD	97.16 ± 38.97
Blood loss (mL), mean ± SD	19.3 ± 14.63
Length of stay (d), mean ± SD	2.79 ± 1.40
Comorbid conditions, *n* (%)	
T2DM	91 (91.1%)
Hypertension	30 (29.7%)
Hyperlipidemia	30 (29.7%)
Obstructive sleep apnea	5 (5%)

The mean operation time for these bariatric surgeries was 97.16 ± 38.79 min and the blood loss was 19.3 ± 14.63 ml. The length of stay after surgery was 2.79 ± 1.4 days (Table 1). There was no perioperative mortality and only one patient (0.99%) suffered a major complication, as leakage at the gastrojejunal anastomosis, which was managed successfully by laparoscopic reoperation for drainage. None of the patients had an incisional hernia 1 year after surgery.

There were 11 patients (10.89%) in Group I (overweight), 45 (44.56%) in Group II (class I obesity), 26 (25.74%) in Group III (class II obesity), and 19 (18.81%) in Group IV (class III obesity) [14]. Table 2 compares the perioperative findings of the different BMI groups. The operation time was significantly longer in the higher BMI group patients (G I *vs.* G II *vs.* G III *vs.* G IV = 81.36 ± 26.72 *vs.* 91.44 ± 36.16 *vs.* 96.42 ± 36.27 *vs.* 124.42 ± 41.31, *P* < 0.01), especially in Group IV. No significant difference was found among the four groups in LOS (G I *vs.* G II *vs.* G III *vs.* G IV = 2.73 ± 1.05 *vs.* 2.67 ± 1.17 *vs.* 2.58 ± 1.03 *vs.* 3.47 ± 2.16, *P* = 0.15), blood loss (G I *vs.* G II *vs.* G III *vs.* G IV = 11.82 ± 5.34 *vs.* 20.11 ± 13.23 *vs.* 19.04 ± 12.81 *vs.* 22.11 ± 20.67, *P* = 0.29), complications (G I *vs.* G II *vs.* G III *vs.* G IV = 0 *vs.* 1 *vs.* 0 *vs.* 0, *P* = 0.74) or cosmetic score (G I *vs.* G II *vs.* G III *vs.* G IV = 4.73 ± 0.45 *vs.* 4.69 ± 0.51 *vs.* 4.81 ± 0.57 *vs.* 4.58 ± 0.49, *P* = 0.54; Table 2). Almost all the patients were very satisfied with the cosmetic results of their operation.

**Table 2:** Comparisons among the different BMI groups during the perioperative period

Variables	Group I (*N* = 11)	Group II (*N* = 45)	Group III (*N* = 26)	Group IV (*N* = 19)	*P*
BMI (kg/m^2^)	27.04 ± 0.61	30.06 ± 1.41	34.34 ± 1.21	44.70 ± 7.02	< 0.01
Operation time (min)	81.36 ± 26.72	91.44 ± 36.16	96.42 ± 36.27	124.42 ± 41.31	< 0.01
Blood loss (mL)	11.82 ± 5.34	20.11 ± 13.23	19.04 ± 12.81	22.11 ± 20.67	0.29
Length of stay (days)c	2.73 ± 1.05	2.67 ± 1.17	2.58 ± 1.03	3.47 ± 2.16	0.15
Complications (*n*)	0	1	0	0	0.74
Cosmetic score	4.73 ± 0.45	4.69 ± 0.51	4.81 ± 0.57	4.58 ± 0.49	0.54

Group I 25 ≤ BMI < 27.5 kg/m^2^

Group II 27.5 ≤ BMI < 32.5 kg/m^2^

Group III 32.5 ≤ BMI < 37.5 kg/m^2^

Group IV BMI ≥ 37.5 kg/m^2^

**Fig. 3:**
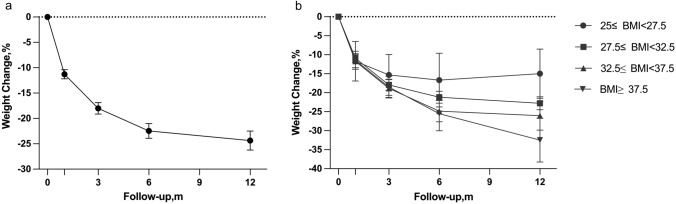
**a** Percentages of weight loss in the 101 patients after surgery. **b** Percentages of weight loss in the four groups after surgery

The follow-up duration was 1 year, with a compliance rate of 92.1% for the completion of this period. Table 3 summarizes the changes in weight loss parameters. Compared with the baseline values, the mean BMI had decreased by 8.67 kg/m^2^ at 12 months (*P* < 0.05). The percentage of total weight loss (%TWL) was 24.37% ± 9.02 (Table 3, Fig. 3, *P* < 0.05). Figure 3 shows the %TWL results for every group. All the patient groups had significant weight reduction at 12 months (G I *vs.* G II *vs.* G III *vs.* G IV = 15.0% ± 8.62 *vs.* 22.8% ± 5.51 *vs.* 26.03% ± 8.81 *vs.* 32.49% ± 10.09, *P* < 0.01). Weight loss at 12 months was significantly different among the four groups. (*P* < 0.01). The patients in Group I had the most weight loss at 6 months (16.67% ± 8.68) and showed a relapse at 12 months.

**Table 3:** Weight and BMI in the preoperative and postoperative periods

	Baseline	1 month	3 months	6 months	1 year
Follow-up	101	93	92	92	93
Weight (kg)	94.20 ± 21.29	83.53 ± 19.29*	76.71 ± 17.45*	72.13 ± 15.18*	69.71 ± 12.77*
BMI (kg/m^2^)	33.59 ± 6.68	29.83 ± 6.12*	27.38 ± 5.57*	25.70 ± 4.50*	24.92 ± 3.80*
%TWL		11.30 ± 4.34 *	18.01 ± 5.36*	22.45 ± 6.99*	24.37 ± 9.02*

*P < 0.05: vs. preoperatively

## Discussion

We reported the 12-month outcomes of 101 patients who underwent STLGB. To our knowledge, this is the first study to demonstrate the application of STLGB and our results showed it to be a safe, effective, and feasible technique. The location of the three ports and every step of the operation was standardized as standardization of the operation is important for quality control, team training, and technology promotion.

Conventional laparoscopic RYGB requires five to seven abdominal incisions, which are associated with poor cosmetic results and the need for strong analgesics postoperatively [15]. In recent years, reports on SILS for bariatric and metabolic surgery have emerged [8, 16–18]. The benefits of SILS over conventional laparoscopic surgery include less pain, earlier recovery, shorter hospital stay, and better cosmesis [6, 9, 19]. However, the application of SILS is restricted by its disadvantages. According to Panagiotis Lainas’ review, only four articles contained data on 196 patients who underwent SILS RYGB over an entire decade [19]. Since the patients in our study were not selected, the proportion of women (60.4%) was lower than that in most SILS bariatric surgery studies [7, 8]. Generally, women are more concerned with cosmetic results after surgery and fewer men have undergone SILS RYGB. The mean age of the patients in the present study was 42.18 ± 9.44 years, which was slightly older than that in the four previous SILS RYGB studies. The mean BMI was 33.59 ± 6.68 kg/m^2^, which was also lower than that in the SILS RYGB studies. Asians, especially Chinese, affected by T2DM, tend to have a lower BMI. Many patients with a BMI < 32.5 kg/m^2^ and T2DM were included in this study.

The mean operating time and blood loss were both less than for SILS RGYB [10, 11, 19, 20]. The symmetric three-port laparoscopic approach and standardization of procedures reduced the operating time and improved the surgery quality. The length of stay was the same as that reported by other studies. A total of 91.1% of patients in our study had T2DM; a much higher proportion than in Huang’s and Chelala’s report [13, 18]. The reason for this is that in our center, patients with T2DM generally select RYGB to control diabetes progression.

Few SILS surgery studies have focused on RYGB results in patients with different obesity classes. To investigate the safety, effectiveness, and feasibility of STLGB, we divided the patients into four groups according to their BMI. We found that operating time increased with a higher BMI because patients with a higher BMI have more visceral fat. The same trend was seen for blood loss and LOS but without significance. Early postoperative complications were rare in every group, with just one case of leakage in group II. Almost all patients in the different BMI groups were very satisfied with the cosmetic results of their operation, like the SILS RYGB patients. According to a previous report, women were more likely than men to choose SILS bariatric surgery [18]. In contrast, regardless of gender or BMI, STLGB was suitable.

Few SILS surgery studies have reported weight loss outcomes. We investigated short-term weight loss after STLGB and noted that significant but not excessive weight loss was achieved in all patients. Some RCT studies have reported mid-term %TWL after RYGB of 22–26% [21–23]. We attributed the variation in %TWL in the different studies to the fact that the baseline weight and BMI levels varied greatly. Jia’s study confirmed similar results in Chinese T2DM patients with different BMI obesity classes [24]. We also observed that weight loss was higher in the higher baseline BMI groups. The %TWL results in our group IV were similar to those reported by Sjostrom et al. [25]. In our study, the %TWL was 24.37% ± 9.02 and the BMI was 24.92 ± 3.80 1 year after surgery, with the mean BMI decreasing by 8.67 kg/m^2^ from the baseline. These results were comparable to those in the SILS RYGB studies [1, 21, 24]. The weight of our Group I patients decreased over 6 months and then rebounded slightly thereafter. Similar results were reported by Zhu [25].

Although the SILS approach to RYGB has been tried by several surgeons, it is not performed widely [9, 10, 13, 18, 26]. There are several reasons limiting the application of SILS RYGB. First, SILS has a critical learning curve caused by the loss of triangulation; thus, SILS RYGB is extremely difficult for new surgeons and only able to be mastered after extensive experience in traditional RYGB surgery. Hence, it is not feasible for low-volume bariatric centers. Second, many specific instruments are needed for SILS, such as multichannel trocars, long flexible graspers, and 5 mm optical scopes with or without flexible goosenecks. Third, costs are much higher for SILS because of the special instruments needed. The literature fails to provide data or evidence on the cost of the procedure. In the report of Chelala and colleagues, an extra 1200 US dollars was expended during the hospitalization of SILS patients [18]. Finally, SILS is not suitable for all patients. Patients with these limiting factors are not candidates: superobese patients with a BMI greater than 50 kg/m^2^, those with left liver hypertrophy (LLH), those with a xiphoid-umbilicus distance (XUD) greater than 28 cm, and those who have had previous epigastrium surgery [18, 27]. In Dagher’s report, although single-port laparoscopic sleeve gastrectomy (SPSG) was a routine procedure in their center, 7.8% of patients still needed additional ports [28]. For superobese patients, approximately 19.3% needed extra ports for the procedure to be completed [29]. It is almost impossible for SILS RYGB to become a routine procedure. In contrast, STLGB rarely needs extra ports. Saber et al. reported a technique of three trocar laparoscopic RYGB, which is similar to the technique of transumbilical 2-site laparoscopic RYGB reported by Lee [10, 30]. This has not been used widely.

STLGB has many advantages over SILS RYGB. First, it is easier for beginners to master and for adoption in low-volume centers. Second, as no extra instruments are needed, it is more cost-effective. Third, it is very safe and beneficial for utilization with the standardization of procedures. Finally, there are almost no limitations in patient selection. Thus, STLGB has become a routine procedure in our center. Despite three ports being required for STLGB, the total length of the incisions is no longer than that of those in SILS RYGB [11]. Most patients were satisfied with their cosmetic results.

The limitations of our study include its retrospective nature and the effect of this surgery on quality of life. Prospective cohort studies and analyses of the quality of life are required and will be conducted. Moreover, as cost-effectiveness is becoming increasingly important in the context of medical insurance, evaluating the cost of this surgery in detail is necessary.

## Conclusion

We developed a symmetric three-port technique for complex bariatric surgery. STLGB has been proven to be easy, safe, and feasible. This approach is suitable for all kinds of patients. Moreover, it is reproducible with standardization of the procedure in low-volume centers. The potential benefits and limitations of the symmetric three-port laparoscopic approach in bariatric surgery require further evaluation by large, prospective studies with long-term follow-up.
